# Differences of Anxiety and Depression in Dry Eye Disease Patients According to Age Groups

**DOI:** 10.3389/fpsyt.2022.930714

**Published:** 2022-07-13

**Authors:** Zhanglin Liu, Shengshu Sun, Xiaowen Sun, Yuan Wu, Yue Huang

**Affiliations:** ^1^Tianjin Medical University Eye Hospital, Eye Institute and School of Optometry, Tianjin, China; ^2^Tianjin Key Laboratory of Retinal Functions and Diseases, Tianjin, China; ^3^Tianjin Branch of National Clinical Research Center for Ocular Disease, Tianjin, China; ^4^Department of Ophthalmology, People’s Hospital of Rizhao, Rizhao, China

**Keywords:** dry eye disease, different age, sleep disorder, anxiety, depression

## Abstract

This study aimed to investigate the association between dry eye disease (DED) and DED-related anxiety and depression tendencies, as well as the risk factors for anxiety and depression in patients with DED of different age groups. This was a cross-sectional study involving 160 patients with DED and 80 healthy individuals aged 20–65 years. All participants completed the investigation of the demographic characteristics, the Hospital Anxiety Depression Scale (HADS), the Ocular Surface Disease Index (OSDI) questionnaire, the Standard Patient Evaluation of Eye Dryness (SPEED) questionnaire, and underwent objective clinical eye examinations. In patients aged 20–40 years, anxiety and depression scores were correlated with OSDI, sleep disorders, and Best Corrected Visual Acuity (BCVA). In patients with DED aged 41−65 years, anxiety scores were correlated with sleep disorders, the level of DED impact on life and work, and the severity of DED. Depression scores were correlated with sleep disorders and the severity of DED. The results indicated that the tendency for anxiety and depression was closely associated with DED and sleep disorders. Moreover, the factors affecting anxiety and depression in patients varied with age.

## Introduction

Dry eye disease (DED) is “a multifactorial disease of the ocular surface characterized by a loss of homeostasis of the tear film and accompanied by ocular symptoms, in which tear film instability and hyperosmolarity, ocular surface inflammation and damage, and neurosensory abnormalities play an etiological role, as defined by the second report of the Tear Film and Ocular Surface Society, TFOS DEWS II (1–3). DED is characterized by diminished production or increased evaporation of tears (non-Sjögren’s syndrome) or by a systemic immunologic disorder (Sjögren’s syndrome), which causes insufficient moisture production in the salivary and lacrimal glands.

The worldwide prevalence of DED is approximately 5.5−33.7% (4–6). With the prolonged use of electronic devices and increasing environmental pollution, the number of patients with DED is rising. DED has become a common and important ocular surface disease that endangers public health and affects the quality of life. The discomfort caused by DED largely affects the physical, psychological, and social functions of patients with DED. DED can lead to a variety of psychological disorders, such as anxiety, depression, fatigue, sleep disorders, and lyrical disorders (6–10). Anxiety and depression are the most common psychological disorders among patients with DED (11, 12). Some of the findings are that the prevalence of anxiety in patients with DED is approximately 39–63.6% and the prevalence of depression in patients with DED is approximately 25−53.7% (4, 7, 13, 14).

To the best of our knowledge, studies on the association between DED and anxiety and depression, such as Sjogren’s syndrome patients or Sjogren’s syndrome patients, and study participants with non-Sjogren’s syndrome, are scarce. Sjogren’s syndrome is a systemic immune disease that tends to cause anxiety and depression among patients. It accounts for a small percentage of daily patients with DED, and patients with non-Sjogren’s syndrome account for the majority of patients with DED. The pathophysiologic changes of DED may occur and may differ between young and elderly patients. Studies have shown that patients with DED aged 20–41 years express more dry eye discomfort symptoms than elderly patients (15). Furthermore, according to various types of research, the age of perimenopausal women around the world is about 40–60 years old (16, 17). Therefore, we divided the patients into two groups, and the age of 40 years was chosen as the cut-off. The link between age-related changes and psychological changes in patients with DED remains unclear. Investigating the association between DED and anxiety and depression in non-Sjogren’s syndrome patients of different ages can timely eliminate the hidden dangers of mental illness in patients with DED and can also control the situation of patients with DED. Simultaneously, attention should be paid to the treatment of DED and the factors that lead to DED.

## Materials and Methods

### Study Design

Eligible participants completed several questionnaires between October 2021 and February 2022 to assess their psychological status and dry eye symptoms. These included the Hospital Anxiety Depression Scale (HADS), the Ocular Surface Disease Index (OSDI), and the Standard Patient Evaluation of Eye Dryness (SPEED). Several clinical eye examinations were also conducted. The study design was approved by the Medical Ethics Committee of the Tianjin Medical University Eye Hospital. The procedures used in this study adhered to the tenets of the Declaration of Helsinki, and informed consent was obtained from all participants included in the study. Dry eye tests were performed on both eyes, and data from the left eye were used for statistical analysis. The questionnaires were self-administered.

### Study Population

#### Inclusion and Exclusion Criteria

The criteria for the DED group included non-Sjogren’s syndrome patients with DED seen at the Tianjin Medical University Eye Hospital (160 cases), aged 20−65 years old. Patients with DED were diagnosed according to the definitions of the TFOS DEWS II. DED diagnostic criteria included patients who complained of subjective symptoms, such as eye dryness, foreign body sensations, burning sensations, fatigue, discomfort, redness, and fluctuating visual acuity. Those with the Chinese Dry Eye Questionnaire scores ≥7 or OSDI scores ≥13, and patients with fluorescein tear break-up time (FBUT) ≤5 s, non-invasive keratograph tear break-up time (NIBUT) <10 s, Schirmer I test (SIT) ≤ 5 mm/5 min were diagnosed with DED. Patients with DED-related symptoms and a score of ≥7 on the China Dry Eye Questionnaire or ≥13 on the OSDI, whereas patients with FBUT >5 s and ≤10 s or NIBUT of 10–12 s and SIT > 5 mm/5 min and ≤10 mm/5 min were examined by fluorescein sodium staining of the cornea (FL), and positive staining (≥5 points) to obtain the diagnosis of DED.

The criteria for the control group included: patients with non-DED (80 cases), aged 20−65 years, attending the Tianjin Medical University Eye Hospital. Patients without DED were diagnosed according to the definitions in TFOS DEWS II. All participants had to be fluent in Chinese, willing and able to complete a series of questionnaires without much assistance, and willing to perform clinical eye examinations as part of the study.

The exclusion criteria for participation in the study included: patients who have used eye medication, such as artificial tears, on the day of the examination; corneal contact lens use within 3 months or eye surgery within 6 months; ocular anatomy or innervation abnormalities (asymptomatic or mildly symptomatic, but with significant tear film function or ocular surface damage); patients with ocular inflammation and other eye diseases not caused by DED; suffering from systemic immune diseases, such as hyperthyroidism, hypertension, diabetes mellitus, rheumatism, and Sjogren’s Syndrome; use of anti-anxiety drugs, such as benzodiazepines diazepam, lorazepam, alprazolam, and oxazepam; use of antidepressants, such as selective serotonin reuptake inhibitors: fluoxetine, paroxetine, sertraline, fluvoxamine, and tricyclic antidepressants: doxepin, amitriptyline, promethazine, and clomipramine, allergic to fluorescein sodium; and pregnant and lactating women, hormone replacement therapy patients, and menopausal women.

### Survey Instrument

The participants completed a standardized assessment of anxiety and depression using the HADS questionnaire. The HADS is a 14-item, four-point Likert scale that contains two subscales (18). Seven items in the scale assess anxiety status (e.g., I feel nervous (or painful), or I am full of worries inside), the other seven items assess depression status (e.g., I am still interested in things that used to interest me or I can see the good side of things). The options for each question were rated as follows: 0-not at all; 1-sometimes; 2-often; and 3-always. The total score area of the anxiety and depression questionnaire is: 0−7, asymptomatic; 8−10, suspicious; and 11−21, definitely present. This scale was used to assess DED-related anxiety and depression.

The OSDI and SPEED questionnaires were used to complete a standardized assessment of ocular discomfort (19, 20). The OSDI, developed by Allergan Inc., is a 12-item patient-reported outcome questionnaire designed to quantify ocular disability due to DED. It focuses on evaluating the common symptoms of DED and their frequency, and can assist in grading the severity of DED, including the frequency of recall of information within 1 week of DED symptoms (photophobia, foreign body sensation, pain or soreness, and blurred or decreased vision), restrictions on daily activities (reading, watching TV, working on a computer or cell phone, or driving at night), or the effects of environmental triggers (wind, low humidity, and air conditioning). Each question is rated as 0-does not happen; 1-sometimes happens; 2-half of the time; 3-most of the time; and 4-always happens. The total score of the OSDI questionnaire equals the sum of scores × 25/number of questions answered. The score ranges from 0−100, 13–22 is mild dry eye; 23−32 is moderate dry eye; and 33−100 is severe dry eye. The SPEED, developed by Tear-Science, Inc., is an 8-item questionnaire that focuses on the correlation between symptoms and risk factors for DED. It is suitable for the epidemiological investigation of DED and the symptom assessment of MGD-related DED. It includes both DED symptoms (dry, itchy, or foreign body sensation; soreness; irritation or tearing; and visual fatigue) and severity. Each question is rated as: 1-sometimes occurs; 2-often occurs; and 3-always occurs. Severity was rated for each question (0-no effect; 1-slight discomfort; 2-discomfort does not affect life; 3-discomfort affects life; and 4-unbearable cannot live normally). Both questionnaires provide valid assessments of DED symptoms and are consistent with the evaluation of DED symptoms. Combining the two can provide a more accurate and comprehensive assessment of symptoms and evidence for the diagnosis of DED.

### Clinical and Socio-Demographic Measures

Participants’ sociodemographic data were collected, such as name, gender, age, educational background, and sleep status (sleep duration > 6 h, number of awakenings > 2 times, and time to fall asleep > 30 min), frequency of sleeping later than 24:00, length of daily visual terminal use (television, smart phone, and computer), level of discomfort affecting life and work (mild, moderate, or severe), DED discomfort duration, level of concern about discomfort (all the time or when it is severe), and smoking or drinking status.

Eye clinical examinations included: best corrected visual acuity (BCVA) (Fractional Recording Method), intraocular pressure (IOP), non-invasive keratograph tear meniscus height (NIKTMH), NIBUT, FBUT, FL (12-Point Method), lipid layer thickness (LLT), partial blink rate (PBR), and the SIT. Each examination was performed by the same ophthalmologist. IOP was measured by the Topcon CT-800 computerized tonometer (Topcon, Japan). A non-invasive ocular surface analyzer (Keratograph 5M, Oculus, Germany) was used to measure the NIKTMH and NIBUT. The measurements were performed in a dark room. The time and location of the first tear film break-up were recorded by an infrared camera system. A fluorescein impregnated strip (Jingming, Tianjin, China) wetted with non-preservative saline solution was placed in the lower conjunctival sac and the patient was asked to blink several times. Using the cobalt blue filter and slit-lamp microscopy, the time required to observe the first area of tear film break-up after a complete blink was recorded as the TBUT. Each examination was repeated three times and the average of the measurements was calculated. FL was observed through a slit-lamp with a cobalt blue filter and divide the cornea into four quadrants. Each quadrant was graded as 0 (no staining), 1 (1–30 punctate staining), 2 (more than 30 unfused punctate staining), and 3 (fused punctate staining, corneal filaments, and ulcers). An ocular surface interferometer (Lipiview, TearScience, America) was used to measure the LLT and PBR. The measurements were performed in a dark room. The patient was asked to look at the indicator light and blink at a habitual frequency. The SIT without topical anesthesia was performed by inserting a sterile Schirmer test strip (Jingming, Tianjin, China) into the inferior fornix at the junction of the middle and lateral third of the lower eyelid margin with the eye closed for 5 min.

### Statistical Analysis

Statistical analysis was performed using SPSS for Windows (version 26.0; SPSS, Chicago, Illinois, United States) and reported as means ± standard deviation (SD) or medians. The Gaussian distribution of the parameters was tested using the Shapiro–Wilk test. To assess statistical differences, we used the Student *t*-test and Mann–Whitney *U*-test for continuous variables, and the Fisher exact test and chi-square test for categorical variables. A Bonferroni corrected *post hoc* test was conducted to adjust the observed significant level for multiple comparisons. The association between variables was examined by applying the Spearman rank correlation test and was expressed as the Spearman correlation coefficient. We used multinomial linear regression analysis to investigate the risk factors for the development of anxiety and depression. The statistical significance was defined as a two-tailed *p*-value < 0.05.

## Results

The demographic data are presented in Table 1. The study population included 160 patients with DED and 80 control participants and was divided into four groups by age range (Table 1). The majority of patients with DED were women (75 and 73.75%) and had poor sleep status (55 and 67.5%) among patients aged 20–40 and 41–65 years, respectively. More than half of the patients experienced discomfort lasting 6 months or more. In patients aged 20−40 years, more than half of the patients had a bachelor’s degree or higher educational level (83.75%), and in patients aged 41–65 years, more than half of the patients had an education level below a bachelor’s degree (55%). Late sleep (57.5 vs. 27.5%) and visual terminal use (8 vs. 6 h) occurred more frequently in patients aged 20−40 years than patients in the ages of 41−65 years. There was no statistically significant difference between the 20−0 year aged DED group and the control group for late sleep (*p* = 0.300), and the length of use of visual terminals showed no statistically significant difference in the patients with DED aged 41−65 years and the control group (*p* = 0.765). In addition, sex showed no significant difference between the two age groups (*p* > 0.05). In the age 20−40 year group, 42 patients (52.5%) felt that the discomfort had a moderate to severe impact on their life or work, whereas in the age 41−65 year group, this value was 37 cases (46.25%). There were 34 patients (42.5%) who were always concerned about discomfort in the age 20−40 year group, compared with 33 patients in the 41−65 year age group (41.25%) (Table 1).

**TABLE 1:** Demographic characteristics of patients with or without dry eye disease (DED).

Variables	Group with DED	Control group	*P*	Group with DED	Control group	*P*
	(*N* = 80)	(*N* = 40)		(*N* = 80)	(*N* = 40)	
Range of age (years)	**20–40**	**20–40**		**41–65**	**41–65**	
Age,years^a^	30 (27–35)	27 (25.25–30)	**0.034**	50 (45–56)	46 (44–49.75)	**0.003**
**Gender, *n* (%)**						
Male	20 (25)	12 (30.0)	0.559	21 (26.25)	14 (35.0)	0.320
Female	60 (75)	28 (70.0)		59 (73.75)	26 (65.0)	
**Level of education, *n* (%)**						
Below bachelor’s degree	13 (16.25)	3 (7.5)	0.184	43 (53.8)	13 (32.5)	**0.028**
Undergraduate or higher	67 (83.75)	37 (92.5)		37 (46.2)	27 (67.5)	
Sleep disorders*, *n* (%)	44 (55)	6 (15.0)	**0.000**	54 (67.5)	4 (10.0)	**0.000**
**Frequency of sleep late, *n* (%)**						
None	34 (42.5)	21 (52.5)	0.300	58 (72.5)	20 (50.0)	**0.015**
More than three times per weeks	46 (57.5)	19 (47.5)		22 (27.5)	20 (50)	
Length of visual terminals use, hours/d^a^	8 (5-10)	6 (5-7)	**0.044**	5 (4–8)	5 (4–6)	0.765
**Level of impact on life and work, *n* (%)**						
None or mild	38 (47.5)	40 (100)	**0.000**	43 (53.75)	40 (100)	**0.000**
Moderate	10 (12.5)	0		13 (16.25)	0	
Severe	32 (40)	0		24 (30)	0	
Discomfort duration, months^a^	6 (1–21)	0	**0.000**	6 (2–24)	0	**0.000**
**Level of concern about discomfort, *n* (%)**						
Always pay attention	34 (42.5)	0	**0.000**	33 (41.25)	0	**0.000**
In case of severe discomfort	46 (57.5)	0		47 (58.75)	0	
**Smoking and drinking, *n*%**						
None	67 (83.75)	30 (75)	0.251	69 (86.25)	33 (82.5)	0.862
Yes	13 (16.25)	10 (25)		11 (13.75)	7 (17.5)	

*Sleep disorders, Sleep duration >6 h, Number of awakenings >2 times, Time to fall asleep >30min.

*Significant values are shown in bold. P-values < 0.05 were considered significant.*

*^a^Median (P25–P75).*

As presented in Table 2, patients had poorer best-corrected visual acuity than the control group. Patients with DED had shorter tear break-up times (NIBUT and FBUT). In the comparison of the four groups, partial blinks were more prevalent in patients with DED than those without DED (0.84 vs. 0.5; *p* = 0.005, 0.8 vs. 0.5; *p* = 0.038). The LLT of DED participants was also significantly thinner than that of the control group (72 vs. 100; *p* = 0.001, 84 vs. 100; *p* = 0.025). With regard to comparisons between the projects of the DED group and the control group, the results show a statistically significant difference, except for IOP (*p* > 0.05).

**TABLE 2:** Ophthalmic findings in dry eye disease (DED) and control group.

Ophthalmic findings	Group with DED	Control group	*P*	Group with DED	Control group	*P*
	(*N* = 80)	(*N* = 40)		(*N* = 80)	(*N* = 40)	
Range of age	20–40	20–40		41–65	41–65	
BCVA	0.94 ± 0.21	1.00 ± 0.09	**0.027**	0.92 ± 0.21	1.00 ± 0.03	**0.001**
Intraocular pressure (mmHg)	14.08 ± 3.02	14.01 ± 2.37	0.900	13.46 ± 3.21	14.08 ± 2.35	0.592
NIKTMH (mm)^a^	0.24 (0.18–0.29)	0.23 (0.21–0.30)	**0.035**	0.21 (0.17–0.26)	0.23 (0.21–0.28)	**0.018**
NIBUT (s)^a^	5.18 (3.71–7.25)	10.63 (8.68–13.23)	**0.000**	5.10 (3.73–6.80)	10.50 (8.38–12.90)	**0.000**
FBUT (s)^a^	4 (3–6)	8 (6–10)	**0.000**	5 (3.08–6)	8 (6–10)	**0.000**
Fluorescein staining score^a^	0 (0–2.75)	0	**0.000**	0 (0–2)	0	**0.000**
LLT (nm)^a^	72 (53.5–100)	100 (88-100)	**0.001**	84 (56–100)	100 (88–100)	**0.025**
Partial blink rate^a^	0.84 (0.40-1)	0.5 (0.33-1)	**0.005**	0.8 (0.43–1)	0.5 (0.31–1)	**0.038**
Schimer I Test (mm/5min)^a^	10 (5–15)	13 (12–14)	**0.024**	9 (5–13.75)	13 (12–14)	**0.000**

*BCVA, best corrected visual acuity; LLT, lipid layer thickness; NIKTMH, non-invasive keratograph tear meniscus height; NIBUT, non-invasive break-up time; FBUT, fluorescein break-up time.*

*Significant values are shown in bold.P-values < 0.05 were considered significant.*

*^a^Median (P25–P75).*

**FIGURE 1:**
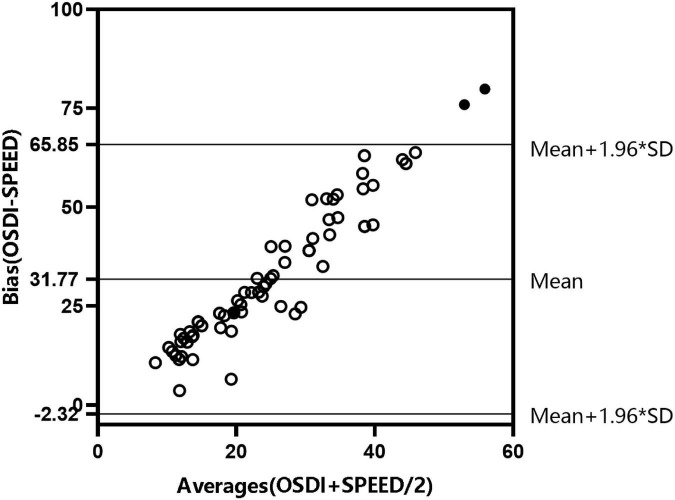
Consistency test of Ocular Surface Disease Index (OSDI) and Standard Patient Evaluation of Eye Dryness (SPEED) questionnaire scores in patients with dry eye disease (DED) aged 20–40.

**FIGURE 2:**
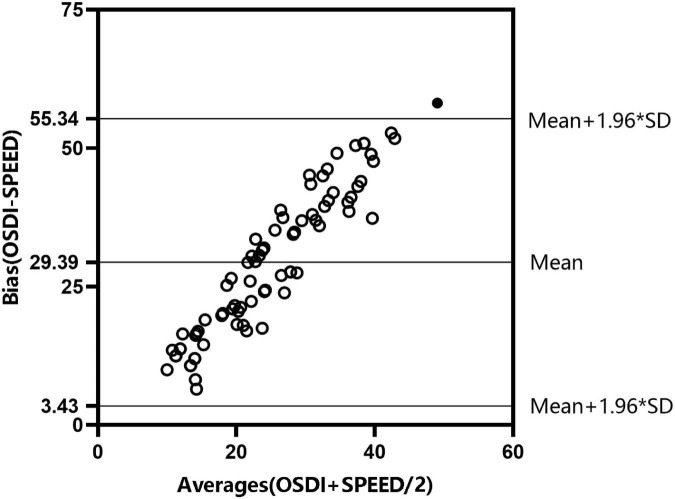
Consistency test of Ocular Surface Disease Index (OSDI) and Standard Patient Evaluation of Eye Drynes (SPEED) questionnaire scores in patients with dry eye disease (DED) aged 41−65.

To accurately evaluate patients’ discomfort symptoms, we used two DED symptom scales as shown in Figures 1 and 2. The results showed that the two scales were consistent. According to the findings of DED symptoms and psychological disturbances (Table 3), the median OSDI and SPEED scores were higher among patients with DED aged 41−65 years compared with those aged 20–40 years (37.50 vs. 38.77; 8 vs. 10). Anxiety and depression scores were higher in patients aged 20−40 years than in those aged 41–65 years and the control group (*p* = 0.000).

**TABLE 3:** Dry eye symptoms and psychological disturbances.

Variables	Group with DED	Control group	*P*	Group with DED	Control group	*P*
	(*N* = 80)	(*N* = 40)		(*N* = 80)	(*N* = 40)	
Range of age	**20–40**	**20–40**		**41–65**	**41–65**	
OSDI scores^a^	37.50 (22.50–56.36)	0	**0.000**	38.77 (27.87–53.46)	0	**0.000**
SPEED scores^a^	8 (5–11)	0	**0.000**	10 (8–13)	0	**0.000**
Anxiety scores^a^	4 (2–7)	0	**0.000**	3 (1–4.75)	0	**0.000**
Depression scores^a^	3 (1.25–6)	0	**0.000**	2 (1–4)	0	**0.000**

*Significant values are shown in bold.P-values < 0.05 were considered significant.*

*OSDI, Oscular Surface Disease Index; SPEED, Standard patient evaluation of eye dryness.*

*^a^Median (P25–P75).*

As presented in Table 4, anxiety scores were found to be correlated with age (*r* = –0.224; *p* = 0.046), sleep disorders (*r* = 0.470; *p* = 0.000), frequency of sleep late (*r* = 0.256; *p* = 0.022), discomfort duration (*r* = 0.357; *p* = 0.001), level of impact on life and work (*r* = 0.342; *p* = 0.002), and depression scores were found to be correlated with sleep disorders (*r* = 0.434; *p* = 0.000) and discomfort duration (*r* = 0.453; *p* = 0.000) in patients with DED aged 20−40 years. In patients aged 41−65 years, anxiety scores were found to be correlated with sleep disorders (*r* = 0.382; *p* = 0.000), the level of impact on life and work (*r* = 0.491; *p* = 0.000), and the level of concern about discomfort (*r* = 0.387; *p* = 0.000), and depression scores were found to be correlated with sleep disorders (*r* = 0.457; *p* = 0.000), the level of impact on life and work (*r* = 0.420; *p* = 0.000), and the level of concern about discomfort (*r* = 0.235; *p* = 0.036).

**TABLE 4:** Correlation of demographic information with psychological disturbances for dry eye disease (DED) patients.

Range of age	20–40				41–65			
	Anxiety scores		Depression scores		Anxiety scores		Depression scores	
	*r*	*P*	*r*	*P*	*r*	*P*	*r*	*P*
Age	**0.224**	**0.046**	–0.059	0.61	0.122	0.282	0.079	0.487
Gender	0.168	0.137	–0.146	0.2	0.087	0.442	0.004	0.971
Level of education	0.085	0.451	–0.108	0.34	–0.202	0.072	–0.183	0.104
Sleep disorders	**0.470**	**0.000**	**0.434**	**0.000**	**0.382**	**0.000**	**0.457**	**0.000**
Frequency of Sleeping later than 24:00	**0.256**	**0.022**	0.083	0.47	0.008	0.944	–0.030	0.794
Length of visual terminals use	0.126	0.264	–0.114	0.31	–0.131	0.246	–0.208	0.064
Discomfort Duration	**0.357**	**0.001**	**0.453**	**0.000**	–0.067	0.555	0.058	0.611
Level of impact on life and work	**0.342**	**0.002**	0.195	0.08	**0.491**	**0.000**	**0.420**	**0.000**
Level of concern about discomfort	0.189	0.092	0.111	0.33	**0.387**	**0.000**	**0.235**	**0.036**
Smoking and drinking	–0.130	0.252	–0.052	0.64	–0.168	0.136	–0.220	0.164

*Sleep disorders, Sleep duration >6 h, Number of awekenings > 2 times, Time to fall asleep > 30mins.*

*Significant values are shown in bold. P-values < 0.05 were considered significant.*

As shown in Table 5, anxiety scores were correlated with OSDI scores (*r* = 0.579; *p* = 0.000), SPEED scores (*r* = 0.329; *p* = 0.003), BCVA (*r* = –0.418; *p* = 0.002), FL (*r* = 0.246; *p* = 0.028), whereas depression scores were correlated with OSDI scores (*r* = 0.503; *p* = 0.000), SPEED scores (*r* = 0.262; *p* = 0.019), and BCVA (*r* = –0.393; *p* = 0.000). In patients aged 41−65, anxiety scores were correlated with OSDI scores (*r* = 0.324; *p* = 0.003), SPEED scores (*r* = 0.239; *p* = 0.033), NIKTMH (*r* = –0.351; *p* = 0.001), FL (*r* = 0.282; *p* = 0.011), and depression scores were found to be correlated with OSDI scores (*r* = 0.377; *p* = 0.001), SPEED scores (*r* = 0.307; *p* = 0.006), NIKTMH (*r* = –0.342; *p* = 0.002), and FL (*r* = 0.221; *p* = 0.049).

**TABLE 5:** Correlation of dry eye symptoms and ophthalmic findings with psychological disturbances.

Range of age	20–40				41–65			
	Anxiety scores		Depression scores		Anxiety scores		Depression scores	
	*r*	*P*	*r*	*P*	*r*	*P*	*r*	*P*
OSDI Scores	**0.579**	**0.000**	**0.503**	**0.000**	**0.324**	**0.003**	**0.377**	**0.001**
SPEED Scores	**0.329**	**0.003**	**0.262**	**0.019**	**0.239**	**0.033**	**0.307**	**0.006**
BCVA	**−0.418**	**0.002**	**−0.393**	**0.000**	0.220	0.051	0.162	0.151
Intraocular pressure(mmHg)	0.207	0.065	0.112	0.321	0.197	0.080	0.030	0.790
NIKTMH(mm)	0.182	0.106	0.040	0.725	**−0.351**	**0.001**	**−0.342**	**0.002**
NIBUT(s)	–0.035	0.756	–0.187	0.097	0.070	0.539	0.071	0.534
FBUT(s)^a^	–0.066	0.561	–0.110	0.331	–0.101	0.372	–0.004	0.970
Fluorescein staining scores	**0.246**	**0.028**	0.042	0.710	**0.282**	**0.011**	**0.221**	**0.049**
LLT(nm)	0.135	0.233	0.056	0.620	–0.055	0.627	–0.119	0.294
Partial blink rate	0.121	0.284	0.092	0.417	–0.104	0.360	–0.054	0.635
Schimer I Test(mm/5min)	0.109	0.335	0.085	0.453	–0.002	0.984	0.022	0.847

*OSDI, Ocular Surface Disease Index; SPEED, Standard Patient Evaluation of Eye Dryness; BCVA, best corrected visual acuity; NIKTMH, non-invasive keratograph tear meniscus height; NIBUT, non-invasive break-up time; FBUT, fluorescein break-up time; LLT, lipid layer thickness. Significant values are shown in bold. P-values < 0.05 were considered significant.*

As presented in Table 6, there were no statistically significant intercorrelation between OSDI and objective signs in patients with DED aged 20–40 years. Furthermore, among other variables, only LLT was correlated with PBR (*p* = 0.014). As presented in Table 7, OSDI scores were significantly correlated with NIKTMH and FL (*p* = 0.003; *p* = 0.019).

**TABLE 6:** Intercorrelation between analyzed variables in patients aged 20–40.

	1	2	3	4	5	6	7	8	
1. OSDI	−								
2. NIKTMH	0.111	−							
3. NIBUT	0.323	0.459	−						
4. FBUT	0.827	0.772	0.148	−					
5. FL	0.627	0.792	0.293	0.663	−				
6. LLT	0.282	0.761	0.126	0.645	0.871	−			
7. PBR	0.279	0.737	0.799	0.984	0.711	0.014*	−		
8. SIT	0.062	0.372	0.704	0.460	0.725	0.966	0.440	−	

*P<0.05. OSDI, Ocular Surface Disease Index; NIKTMH, non-invasive keratograph tear meniscus height; NIBUT, non-invasive keratograph tear break-up time; FBUT, fluorescein tear break-up time; FL, fluorescein sodium staining; LLT, lipid layer thickness; PBR, partial blink rate; SIT, Schirmer I test.

**TABLE 7:** Intercorrelation between analyzed variables in patients aged 41–65.

	1	2	3	4	5	6	7	8	
1. OSDI	−								
2. NIKTMH	0.003*	−							
3. NIBUT	0.723	0.050	−						
4. FBUT	0.452	0.556	0.001*	−					
5. FL	0.019*	0.005*	0.001*	0.001*	−				
6. LLT	0.780	0.878	0.045*	0.011*	0.659	−			
7. PBR	0.670	0.626	0.792	0.854	0.021*	0.090	−		
8. SIT	0.466	0.235	0.019*	0.001*	0.011*	0.137	0.013*	−	

*P<0.05. OSDI, Ocular Surface Disease Index; NIKTMH, non-invasive keratograph tear meniscus height; NIBUT, non-invasive keratograph tear break-up time; FBUT, fluorescein tear break-up time; FL, fluorescein sodium staining; LLT, lipid layer thickness; PBR, partial blink rate SIT, Schirmer I test.

Adjusted for independent variables correlated with anxiety and depression scores, multivariate analyses using multiple linear regression revealed that the OSDI, BCVA, and sleep disorders were risk factors for anxiety and depression. OSDI scores and sleep disorders positively affected anxiety and depression scores, and BCVA negatively affected anxiety and depression scores in patients with DED aged 20–40 years (Tables 8, 9). In patients aged 41−65 years, the risk factors for anxiety were sleep disorders, level of discomfort impact on life and work, and FL. The risk factors for depression were sleep disorders and FL (Tables 10, 11). The sample data were independent, and no multicollinearity exists between the independent variables. The residuals follow a normal distribution.

**TABLE 8:** Multiple linear regression analysis of association between anxiety scores, demographic and dry eye parameters in patients aged 20–40.

Adjusted *R*^2^ = 0.467	*B*	Beta	SE	*t*	*P*
OSDI on anxiety	0.061	0.341	0.023	2.717	**0**.**008**
BCVA on anxiety	–3.496	–0.203	1.610	–2.172	**0**.**033**
Sleep disorders on anxiety	1.612	0.226	0.664	2.426	**0**.**018**
Age on anxiety	–0.048	–0.072	0.060	–0.805	0.432
FL on anxiety	0.155	0.117	0.111	1.395	0.167
Sleep late on anxiety	0.108	0.015	0.615	0.176	0.861
Discomfort duration on anxiety	0.023	0.142	0.015	1.507	0.136
Level of DED discomfort impact on anxiety	0.184	0.048	0.386	0.476	0.636

*OSDI, Ocular Surface Disease Index; BCVA, best corrected visual acuity; FL, fluorescein sodium staining. Significant values are shown in bold. P-values < 0.05 were considered significant.*

**TABLE 9:** Multiple linear regression analysis of association between depression scores, demographic and dry eye parameters in patients aged 20-40.

Adjusted *R*^2^ = 0.383	*B*	Beta	SE	*t*	*P*
OSDI on depression	0.057	0.325	0.020	2.884	**0.005**
BCVA on depression	–3.744	–0.225	1.629	–2.298	**0.024**
Sleep disorders on depression	1.382	0.200	0.688	2.011	**0.048**
Discomfort duration on depression	0.023	0.150	0.015	1.534	0.129

*OSDI, Ocular Surface Disease Index; BCVA, best corrected visual acuity; FL, fluorescein sodium staining. Significant values are shown in bold. P-values < 0.05 were considered significant.*

**TABLE 10:** Multiple linear regression analysis of association between anxiety scores, demographic and dry eye parameters in patients aged 41–65.

Adjusted *R*^2^ = 0.384	*B*	Beta	SE	*t*	*P*
Sleep disorders on anxiety	1.589	0.199	0.784	2.026	**0.046**
FL on anxiety	0.628	0.350	0.187	3.368	**0.001**
Level of DED discomfort impact on anxiety	1.158	0.285	0.485	2.387	**0.020**
NIKTMH on anxiety	–8.140	–0.131	6.282	–1.296	0.199
OSDI on anxiety	–0.018	–0.075	0.028	–0.636	0.527
Level of concern about discomfort on anxiety	0.589	0.077	0.898	0.656	0.514

*OSDI, Ocular Surface Disease Index; FL, fluorescein sodium staining; NIKTMH, non-invasive keratograph tear meniscus height. Significant values are shown in bold. P-values < 0.05 were considered significant.*

**TABLE 11:** Multiple linear regression analysis of association between depression scores, demographic and dry eye parameters in patients aged 41–65.

Adjusted *R*^2^ = 0.362	*B*	Beta	SE	*t*	*P*
Sleep disorders on depression	1.631	0.231	0.707	2.309	**0.024**
FL on depression	0.623	0.391	0.168	3.706	**0.000**
Level of DED discomfort impact on depression	0.840	0.233	0.437	1.922	0.058
NIKTMH on depression	–7.758	–0.141	5.659	–1.371	0.175
OSDI on depression	–0.005	–0.022	0.025	–0.188	0.852
Level of concern about discomfort on depression	–0.283	–0.042	0.809	–0.349	0.728

*OSDI, Ocular Surface Disease Index; FL, fluorescein sodium staining; NIKTMH, non-invasive keratograph tear meniscus height. Significant values are shown in bold. P-values < 0.05 were considered significant.*

## Discussion

In this study, our results provided insight into the association between DED and anxiety and depression. We demonstrated that patients with DED reported higher anxiety and depression scores than those without DED in both age groups. Anxiety and depression scores were correlated with the OSDI and our study evaluating the association between anxiety and depression with DED parameters reported that anxiety and depression were associated with BCVA in patients aged 20−40 years, and anxiety and depression were associated with FL in those patients aged 41−65 years.

There are several possible explanations for these results. First, DED symptoms may induce or increase anxiety and depression symptoms, chronic pain, and impaired visual function, negatively affecting the patient’s daily and quality of life (21). The patient’s BCVA or quality of vision is worse than before, and vision fluctuates frequently. In addition, the need for frequent medical care, instillations of eye drops, and high costs of medical care can also affect social interactions (22). A few previous studies (23–25) showed that the presence of chronic pain is negatively correlated with the performance of daily activities, capacity to work, and emotional well-being. The effects of DED symptoms on many components of daily life may contribute to the development of anxiety and depression in patients with DED. Other relevant studies have also demonstrated an association between anxiety and depression and OSDI (26–28), which is in perfect accordance with the results of our study. Moreover, anxiety or depression may aggravate DED symptoms or lower the threshold for perceiving ocular surface discomfort. An altered psychological status can predispose patients to significantly more eye discomfort (29, 30). Second, inflammation plays a crucial role in the development of DED, and the anti-inflammatory potential of *n*-3 polyunsaturated fatty acids (PUFAs) has been shown to be helpful in alleviating DED symptoms (31). Carisha et al. (32) reported decreased levels of *n*-3 PUFAs in patients with depression. An increased PUFAs *n*-6:*n*-3 ratio in the diet has been suggested as an important cause of the increased incidence of both DED and depression (31, 33). An increased *n*-6:*n*-3 ratio promotes the production of proinflammatory cytokines, such as interleukin (IL)-1, IL-6, and tumor necrosis factor (TNF)-α. These cytokines provoke ocular surface inflammation, resulting in positive fluorescein staining and reduced tear production in patients aged 41−65 years. Therefore, anxiety and depression scores correlated with NIKTMH. Moreover, these cytokines produce and enhance negative mood by affecting neurotransmission and signal transduction (34). Therefore, increased production of inflammatory cytokines may be a cause of the overlap in the pathogenesis of these two diseases. In addition, DED, anxiety, and depression may share a common pathophysiology, and the two diseases share common risk factors, such as being female and experiencing menopause, suggesting that sex hormones are involved in both diseases (28).

In addition, a low correlation between discomfort symptoms and objective signs in patients with DED were presented in our study. Several studies also reported that there was a low correlation between the severity of DED symptoms and clinical diagnostic test results (35, 36). Much of this discrepancy can be explained by the lack of repeatability of the objective tests, with the implication that repeated measures of the same test on the same subjects at different times are not strongly correlated. Thus, objective tests will fail to correlate with each other (37, 38). Another plausible reason for a lack of correlation between clinical tests and subjective symptoms may be the natural variability of the disease process, the subjective nature of reported symptoms by patients, the variability in pain thresholds and cognitive responses about the physical sensations in the eyes (22).

For these reasons, anxiety and depression may lead to a decreased pain threshold and an increased sensitivity to physical sensations (39–42). Studies have indicated that somatization is present in most anxiety and depression patients, which may play a role in exacerbating DED symptoms (43, 44). Increased levels of inflammatory cytokines in blood and tears of patients with anxiety and depression will lead to the activation of the brain cytokine system (45–47). Cytokines actually function as a motivational signal that tells the brain to change the organism’s priorities in the face of the threat represented by pathogens or danger signals. This reorganization of priorities results in changes at the subjective, behavioral, and physiological levels. Therefore, the brain cytokine system can undergo sensitization in response to stimulation (47).

On the other hand, patients with anxiety and depression tend to have sleep disorders (48–50). We found that sleep disorders occurred more frequently among people with DED than among the non-DED population. Sleep disorders correlated with anxiety and depression scores in patients aged 41−65 years. Sleep disorders have been reported to aggravate corneal epithelial defects and reduce tear production (51). Sleep disorders increase cortisol, epinephrine, and norepinephrine levels and reduce parasympathetic nerve activity (52). The lacrimal glands, which secrete tears, are innervated by sympathetic and parasympathetic nerves (53). Additionally, sleep disorders may cause mild activation of the human hypothalamic-pituitary-adrenal axis, leading to diuresis (54, 55). In addition, the circadian rhythm of hormones in the renin–angiotensin–aldosterone system is significantly altered. Therefore, the tear production of patients with anxiety and depression will be reduced, which leads to exacerbating DED symptoms.

We found that anxiety scores correlated with age and the impact of DED on life and work in patients aged 20−40 years. Patients aged 20−40 years spent more time using electronic devices. This may illustrate that most young patients are concerned about eye discomfort and its impact on their work and life. The more discomfort lasts, the more the patient’s work and life are impacted, which explains why the discomfort duration was correlated with anxiety scores. Anxiety and depression were also associated with the impact of DED on life and work rather than the OSDI scores in patients aged 41−65 years. Similarly, anxiety and depression scores correlated with the level of concern about DED discomfort in patients aged 41−65 years. This may indicate that there may be no serious eye discomfort, but most patients aged 41−65 years are still overly concerned about eye damage caused by DED and its impact on work and life. For younger patients, eye discomfort was addressed, along with an appropriate treatment plan for the patient’s work situation. For older patients, more psychological counseling is required, in addition to addressing eye discomfort. Our findings that anxiety and depression were associated with OSDI and BCVA in patients aged 20−40 years and OSDI and FL in patients aged 41−65 years were well confirmed. FL was one of the evaluation indicators used to assess the severity of DED; therefore, it may be stated that the anxiety and depression were associated with the severity of DED.

A complete blink maintains a dynamic balance of tear volume on the ocular surface, which is important for the development and distribution of the lipid layer. Blinking serves a vital function in preserving moisture, the unity of the ocular surface of the lipid layer, and the extension of tear lipids. Increasing the PBR leads to inadequate lipid distribution, which may increase tear evaporation (56, 57). Previous studies have shown that blinking is associated with DED, psychological status, and systemic diseases (58). PB was more prevalent in patients with DED than non-DED, and PB was correlated with DED in our study (57). However, no significant correlation was found between PBR and anxiety and depression scores in our study. Many factors may affect a patient’s blinking, such as psychological, physical, and environmental influences. Thus, there may still be a link between PBR, anxiety, and depressive symptoms, which is worth exploring.

In our study, late sleep and DED discomfort duration were not risk factors for anxiety or depression. However, there was a significant difference between patients with DED and without DED in terms of sleep delay and DED discomfort duration. The observed correlation among sleep delay, DED discomfort duration, and psychological disturbances showed that sleep delay and DED discomfort duration play a critical role in the relationship between DED and psychological disturbances.

Our study also has limitations that need to be considered when interpreting the results. First, although the OSDI and SPEED questionnaires are validated and widely used instruments to evaluate dry eye, they may not accurately identify the DED symptoms of patients with corneal sensation disorders (which might be caused by older age, neurological problems, etc.) (30, 59–61). Therefore, it is advisable to evaluate a patient’s corneal nerve condition using confocal microscopy before assessing the symptoms of DED. Second, due to its cross-sectional nature, this study did not show a causal relationship. It remains unclear whether anxiety or depression is a predisposing factor for DED or vice-versa, although we consider the two conditions to influence each other. Finally, although we controlled for the ratio of male and female patients in each group, sex is to have an effect on DED. Further studies with larger sample sizes using different methods are desirable to investigate the relationship among DED, psychological disturbances, and sleep disorders.

## Conclusion

Our study found that anxiety and depression were associated with DED discomfort, sleep disorders, and partial objective DED parameters in patients of various ages. Attention should be paid to patients of different ages. This study has certain clinical significance and may provide guidance for clinicians to treat patients with DED. Health education and psychological counseling should be provided to patients with DED to prevent excessive psychological stress to better alleviate their DED condition and prevent them from developing psychological issues.

## Data Availability Statement

The raw data supporting the conclusions of this article will be made available by the authors, without undue reservation.

## Ethics Statement

The studies involving human participants were reviewed and approved by the Medical Ethics Committee of Tianjin Medical University Eye Hospital (No. 2021KY (L)-09). The patients/participants provided their written informed consent to participate in this study. Written informed consent was obtained from the individual(s) for the publication of any potentially identifiable images or data included in this article.

## Author Contributions

ZL, SS, XS, and YW: material preparation, data collection, and analysis. ZL: write the first first draft of the manuscript and all authors commented on the previous versions of the manuscript. All authors contributed to the conception and design of the study, and read and approved the final manuscript.

## Conflict of Interest

The authors declare that the research was conducted in the absence of any commercial or financial relationships that could be construed as a potential conflict of interest.

## Publisher’s Note

All claims expressed in this article are solely those of the authors and do not necessarily represent those of their affiliated organizations, or those of the publisher, the editors and the reviewers. Any product that may be evaluated in this article, or claim that may be made by its manufacturer, is not guaranteed or endorsed by the publisher.
